# Audit of Electroconvulsive Therapy Service Provision in Lincolnshire Partnership Foundation Trust: Current Standards and Adherence to National Guidance

**DOI:** 10.1192/bjo.2024.598

**Published:** 2024-08-01

**Authors:** Sandar Kyaw, Ayesha Butt, Anita Priya, Girish Kunigiri, Praveen Kumar

**Affiliations:** Lincolnshire Partnership Foundation Trust, Lincoln, United Kingdom

## Abstract

**Aims:**

To improve the quality of care received by service users of Electroconvulsive Therapy (ECT) treatment in Lincolnshire Partnership Foundation Trust (LPFT) by measuring the compliance of the local ECT clinic in Lincolnshire in accordance with National Institute of clinical excellence guidance and ECT accreditation services standards.

**Methods:**

Pre-audit work up includes consultations with ECT clinic lead and stake holders to ensure ethical and governance standard are met. This audit is conducted with the permission of trust quality and safety team.

Sample population is identified from ECT clinic registry, Lincoln. A total of 10 patients who received ECT treatment between January 2023 and August 2023 are included regardless whether the necessary information is available on the clinical system or not, to minimise selection bias. Retrospective data collection by using Rio electronic case records. Descriptive analysis of data using Microsoft Excel and evaluation of results is based on 3 key domains such as indication, consent process and monitoring.

**Results:**

A total of 10 service users, comprising 30% males and 70% females, underwent treatment in both inpatient (80%) and outpatient (20%) settings, primarily for severe depressive illness. In 70% of cases, a pre-ECT assessment was documented to evaluate potential risks and benefits. The consent procedure was completed by a psychiatrist in 70% of instances. However, ongoing consent was not consistently reviewed at each ECT treatment.

Baseline monitoring using the Clinical Global Impression and Comprehensive Psychopathological Rating Scale was conducted in 20% of cases, with no follow-up assessments performed after each treatment. The Montgomery–Åsberg Depression Rating Scale was employed at baseline for 40% of patients, yet there was no evidence of weekly monitoring. While the Montreal Cognitive Assessment was administered to all patients at baseline, it was not conducted after every four treatments.

Post-ECT follow-up data revealed that less than a quarter of patients underwent clinician reviews. Validated rating scales were utilized in no more than a fifth of patients at both one week and two months after treatment.

**Conclusion:**

The findings suggest the need for improved documentation of the entire consent process and in regularly assessing the ongoing validity of consent. Moreover, there is a need for stronger monitoring at baseline, during, and after ECT treatment. It is recommended to revise the local ECT record pathway by December 2023, with a follow-up re-audit scheduled for March 2024 to evaluate the effectiveness of the implemented changes.

